# Sport Spectatorship and Health Benefits: A Case of a Japanese Professional Golf Tournament

**DOI:** 10.3389/fpsyg.2020.01494

**Published:** 2020-07-21

**Authors:** Yasuhiro Watanabe, Tyreal Y. Qian, Jerred J. Wang, N. David Pifer, James J. Zhang

**Affiliations:** ^1^Department of Sport Business Administration, Hiroshima University of Economics, Hiroshima, Japan; ^2^School of Kinesiology, Louisiana State University, Baton Rouge, LA, United States; ^3^Department of Sport Leadership and Management, Miami University, Oxford, OH, United States; ^4^Department of Kinesiology and Sport Management, Texas Tech University, Lubbock, TX, United States; ^5^Department of Kinesiology, International Center for Sport Management, University of Georgia, Athens, GA, United States

**Keywords:** golf spectators, market demand, length of stay, population health benefits, step counts

## Abstract

It has been well-argued that professional sport or mega sport events could serve as a catalyst for sport participation behaviors through direct and indirect trickle-down effects. However, there is limited research exploring the impact of spectator services during sport events on personal and collective well-being. Elaborating on the social-ecological model and the concept of market demand, this study attempted to fill the gap in the literature by measuring core product (player attraction, event attractiveness, and course characteristics) and peripheral spectator services (event services, event information, event amenity, and parking and transportation) of a professional golf tournament hosted in Japan and examining how these services factors would influence spectators’ length of stay at a golf event, physical activity as measured by step counts, self-rated health, and life satisfaction. Following the administration of a questionnaire to spectators at a Japanese professional golf tournament (*n* = 306) and conducting confirmatory factor analyses (CFA) and structural equation modeling (SEM) analyses, research findings revealed that the tournament-related spectator services would have an influence on physical activity, which could in turn influence self-rated health and subsequently impact life satisfaction. That is, emphasizing the sense of accomplishment fulfilled in sport spectatorship is recommended for the well-being of the spectators. The findings of this study shed light on the significance of promoting golf spectatorship as an effective means to facilitate a healthy lifestyle and in the meantime provide golf event marketers with a unique, positive benefit through which their events can be promoted.

## Introduction

Research on the relationship between spectator sports and population health (e.g., physical activity participation, life satisfaction) has received considerable attention from scholars and practitioners alike in recent years (Banyard and Shevlin, 2001; Frawley and Cush, 2011; Wann et al., 2011; Wang et al., 2013; Elling et al., 2014; Inoue et al., 2015; Aizawa et al., 2018). In fact, to curb rising public health spending, the Japanese government has invested substantial resources in, and directed budgets toward, preventive policies and measures. Nevertheless, the rate of physical inactivity is still rapidly increasing. The number of Japanese adults’ daily walking steps has gradually declined since the late 1990s, and reports have shown that Japanese adults have the highest sitting time per day among the top 20 industrialized countries (Bauman et al., 2011; Inoue et al., 2011). In the wake of a successful bid to host the 2020 Olympic-Paralympic Games in Tokyo, several programs and policies have been established to encourage sport participation and promote physical activity. It has been well-argued that professional sport or mega sport events could stimulate regional economy and revitalize local communities (Japan Sports Agency, 2018; Misener et al., 2015; Weed et al., 2015). Research findings also suggest that mega sport events could serve as a catalyst for sport participation behaviors through direct and indirect trickle-down effects (Misener et al., 2015; Weed et al., 2015; Potwarka and Leatherdale, 2016; Aizawa et al., 2018). However, there are still a limited number of studies exploring how the management and marketing of spectator services during sport events would impact personal and collective well-being (Chelladurai, 2014; Inoue et al., 2015). Despite the upward trend of health promotion campaigns taking place lately in Japan, even less research of this nature can be found. Given the growing health concerns over an aging Japan and the potential of spectator sport events to provide health benefits, it is of great importance for sport management scholars to move this research agenda forward and bridge the gap between spectator services and their capacity to improve population health (Chalip, 2006; Inoue et al., 2015; Warner, 2019).

Golf has long been one of the most popular sport and leisure activities in Asia, especially among middle-aged Japanese. Unlike many other forms of spectator sports, golf spectatorship can potentially be of profound physical and mental benefits. For instance, walking while spectating a golf tournament is considered a good form of health-enhancing physical activity (HEPA) that strengthens bones and muscles with minimal risk of injury (Lyu and Lee, 2013). Many spectators have utilized golf tournaments to acquire health benefits by walking on spectator paths around the courses (Watanabe et al., 2013; Murray et al., 2018). There is a growing recognition of the important roles that spectator services in the form of core and peripheral tournament offerings (Byon et al., 2013) play in golf tournament attendance, such as the scenery and landscapes of golf courses, presence of well-known players, and event operations in the areas of staff performance, concession sales, and facility accessibility (Lyu and Lee, 2013; Watanabe et al., 2013; Watanabe and Zhang, 2019). Arguably, in addition to enhancing golf spectators’ experiences, these tournament services may also provide an opportunity for spectators to engage in HEPA by motivating them to achieve recommended daily step counts and boost their self-rated health (Helliwell, 2003; Huang and Humphreys, 2012; Lyu and Lee, 2013; Murray et al., 2017b).

Even so, little is known as to what and how spectator services could influence golf spectators’ physical activity and well-being variables (i.e., step counts, self-rated health, and life satisfaction). Through a social-ecological perspective, this study attempted to fill the research void by measuring core product and peripheral spectator services of a professional golf tournament hosted in Japan and examining how these services factors would influence spectators’ length of stay, physical activity as measured by step counts, self-rated health, and life satisfaction. In doing so, the current research sought to make two major contributions to the literature. First, this study aimed to provide new evidence for the health benefits accrued from golf spectatorship by clarifying and adding new knowledge to previous research on how sport could be managed and marketed to provide health-related benefits (Inoue et al., 2015, 2017; Murray et al., 2017a, b, 2019). Second, given growing interest in understanding the influence of people’s interactions with physical and sociocultural surroundings on health-related behaviors, as well as inconclusive evidence regarding how tournament services could enhance spectators’ physical activity and subjective well-being (Stokols, 1996; Giles-Corti and Donovan, 2002; Pikora et al., 2003; Sato et al., 2016a, 2019; Inoue et al., 2017; Murray et al., 2019), this study made an initial effort to identify the social and physical environmental elements through which health-related behaviors occurred in golf spectatorship.

## Review of Literature

### Theoretical Background

In the present study, we adopted a social-ecological model of human behavior to conceptually guide our investigation of the social and physical environmental correlates of golf spectators’ well-being outcomes. Social-ecological models have evolved over the past decades and been increasingly recognized due to their relevance to health-related behaviors and ever-increasing applications in research and practice, including but not limited to education, public health, psychology, sociology, and sport management (Stokols, 1996; Sallis et al., 1998, 2006; Pikora et al., 2003; Saelens et al., 2003a, b; Langille and Rodgers, 2010; Sato et al., 2019). The core notion of a social-ecological model is that health-related behaviors are impacted by multiple levels of influences, including factors at the intrapersonal, interpersonal, organizational, physical environmental, community, and public policy levels (McLeroy et al., 1988; Sallis et al., 1998, 2006, 2015). Rather than solely relying on psychosocial models, social-ecological models are favored by public health scholars because of their explicit inclusion of people’s interactions with sociocultural and physical environments that are posited to influence health-related behaviors (Stokols, 1992, 1996; Sallis et al., 2006). Past research has suggested that integration of psychosocial and environmental variables is conducive to explaining health-related behaviors, in particular physical activity (Stokols, 1996; Sallis et al., 1998, 2006; Giles-Corti and Donovan, 2002; Saelens et al., 2003a, b).

**FIGURE 1:**
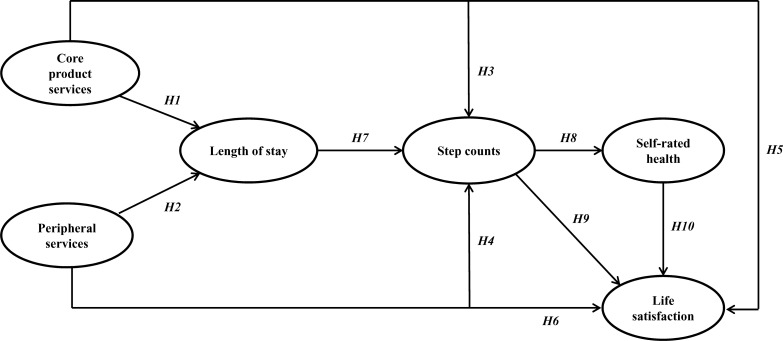
Conceptual model.

Even so, it is worth noting that much of the existing scholarly work has been devoted to examining the influence of inter- and intra-personal variables on people’s well-being (e.g., Sato et al., 2016a, b; Inoue et al., 2017), although a growing body of literature has indicated that physical activity is also influenced by environmental variables (e.g., Brownson et al., 2000; Sallis et al., 2015; Sato et al., 2016a, 2019). Understanding the relative influence of particular physical and social environments on physical activity can help maximize well-being outcomes in those settings (Sallis et al., 1998). Thus, in line with this approach, we posited that delving into the physical and social environmental factors of golf tournaments could add explanatory value provided by intrapersonal and interpersonal factors to a better understanding of how golf tournaments could provide health benefits to spectators (Figure 1).

#### Spectator Services and Length of Stay

Consumer demand is often referred to as market demand that is defined as a consumer’s wants and expectations toward the core and peripheral attributes of a product or service (Zhang et al., 2003; Braunstein et al., 2005; Byon et al., 2013). Previous studies have examined demand factors in various sport and entertainment settings including baseball (Braunstein et al., 2005), basketball (Zhang et al., 2003), action sports (Tsuji et al., 2007), Taekwondo (Kim et al., 2009), golf (Watanabe et al., 2013; Watanabe and Zhang, 2019), Formula 1 (Watanabe et al., 2018), and esports (Qian et al., 2019, in press). Consistent with the social-ecological model of human behavior (Stokols, 1996; Sallis et al., 1998, 2006; Saelens et al., 2003a, b) and the conceptualization of spectator services (Chelladurai, 2014), consumer demand factors for spectator sport can be viewed as social and physical environmental constructs that represent spectators’ evaluation of, and expectation toward, the essential features of a contest, ancillary services, and third place experience.

Sport event spectators primarily attend sport events for the core products, namely, competitive athletic contests, while enjoying peripheral services such as halftime shows and game-day promotions that can only be experienced at the event venue (Mullin et al., 2014; Inoue et al., 2015). Some researchers have noted that provisions of spectator services, including both core and peripheral services, are critical in defining spectators’ experience (Zhang et al., 2003; Tsuji et al., 2007; Byon et al., 2013). In the current research setting, due to the unique nature of golf tournaments (i.e., spectating while walking), the assessment of golf tournament services needs to be approached cautiously. By critically reviewing pertinent sport management and public health literature on this topic, we conceptualized and proposed three subdimensions for core product services and peripheral services, respectively (Hansen and Gauthier, 1993, 1994; Sallis et al., 1998; Saelens et al., 2003a; Byon et al., 2013; Watanabe et al., 2013, 2018). Core product services include player attraction, event attractiveness, and course characteristics, representing the core social and physical environmental appeals that attract spectators to attend a golf tournament (Watanabe et al., 2013; Watanabe and Zhang, 2019). In contrast, peripheral services are reflective of the features of tournament support/operation programs that are closely related to other product functions of a tournament (Zhang et al., 2003). In the current study, peripheral services incorporate event services, event information, event amenity, and parking and transportation, which are instrumental to the overall operational effectiveness of a golf tournament.

Previous research findings indicate that the perceived core product attributes (e.g., the game itself, players’ performances) and event support program features (e.g., the physical environment, event staff, concessions, promotions) might play an important role in influencing spectators’ event satisfaction, desire to stay, and repatronage intentions (Zhang et al., 1995, 2003; Watanabe et al., 2018). For instance, Wakefield et al. (1996) showed that sportscape elements covering a wide spectrum of spectator services influenced football and baseball spectators’ attendance intentions. In the field of tourism and hospitality, Lam et al. (2011) noted that gaming customers who possessed a favorable assessment of the physical environment and casino services would have a higher level of desire to stay and intention to revisit. Similarly, Siu et al. (2012) showed how customers’ evaluation of environmental stimuli could influence their affective responses, revealing the vital role of perceived environmental elements of a convention center in influencing visitors’ satisfaction and desire to stay. Based on these findings, we posited that spectators’ perceived core product and peripheral services would have a positive influence on their length of stay at a golf tournament as the length of stay is mainly dependent on the assessment of spectator services of the tournament (Zhang et al., 2005; Mullin et al., 2014). As such, the following hypotheses were tested.

H1: Core product services would positively correlate with spectators’ length of stay.
H2: Peripheral services would positively correlate with spectators’ length of stay.

#### Spectator Services and Physical Activity

Spectator services by themselves are essential antecedents of sport event consumption; however, they might also be associated with health-related benefits through increased physical activity levels. It has been found that social and physical environments are associated with physical activity such as walking, cycling, and life-space mobility (Giles-Corti and Donovan, 2002; Hoehner et al., 2005; Rantakokko et al., 2015; Chaudhury et al., 2016). Hoehner et al. (2005) assessed the environmental determinants of physical activity among urban adults through their perception of five major environmental dimensions: land use (e.g., destinations within walk distance), recreational facilities (e.g., park, trail, fitness facility), transportation environment (e.g., sidewalks present), aesthetics (e.g., neighborhood pleasant), and social environment (e.g., neighbors physically active). They found that recreational physical activity was positively associated with perceived access to recreational facilities while transportation-related physical activity was positively associated with number of destinations and public transit, yet negatively associated with perceived neighborhood aesthetics. Saelens et al. (2003a) found that environmental variables such as land use mix could add to variance accounted for beyond sociodemographic predictors of walking/cycling for transport in local neighborhoods. They also called for an evaluation of additional environmental variables such as neighborhood aesthetics and topography that might be related to physical activity. Rantakokko et al. (2015) identified perceived environmental facilitators outdoors such as green area, familiar environment, appealing scenery, resting places by the walking route, walkways without steep hills, good quality walkways, other people outdoors who motivate, and close-by services (i.e., shops, marts), showing that people who reported four to seven facilitators were less life-space restricted compared with those who reported three or fewer facilitators.

In a similar vein, the social and physical environmental elements offered by a professional golf tournament might also correlate with spectators’ physical activity. Since golf spectatorship is characterized by long outing hours as opposed to many other forms of team sport events, it should not be too surprising that the health benefits are generally greater for spectators who walk on a course in contrast to players who usually ride on a golf cart (Kras and Larsen, 2002; Watanabe et al., 2013; Murray et al., 2018). Murray et al. (2017b) exhibited that 82.9% of golf spectators reached their daily recommended amount of physical activity when measured by step counts (>7,500 steps). Ostensibly, spectators might unconsciously engage in moderate physical activity by walking behind beloved or famous professional golf players; at the same time, a good quality walking path on the golf course and a pleasant atmosphere of the tournament might provide spectators incentive to explore the course and walk more. Other peripheral services such as event amenity and event services might also impact spectators’ desire to walk as supported by the spillover effect of customer evaluation of ancillary services that could sometimes go beyond the domain or range of the original behavioral area (Zeithaml et al., 1996; Athanassopoulos et al., 2001; Szymanski and Henard, 2001). For instance, attendance at sport events may promote stronger intentions to exercise among participants who are more satisfied with their event experience (Funk et al., 2011a, b); Funk et al. (2011b) found that when people were satisfied with their experience in a participant sport event, they were more likely to attend future events and intended to engage in more physical activities afterward. This makes improving core and peripheral spectator services a critical objective for event organizers as they should strive for offering high-quality spectator services and meeting consumer wants and expectations to not only promote tournament attendance and golf consumption but also nurture health benefits and fitness values (Wakefield and Blodgett, 1996; Watanabe and Zhang, 2019). Subsequently, the following hypotheses were posited.

H3: Core product services would positively correlate with step counts during the tournament.
H4: Peripheral services would positively correlate with step counts during the tournament.

#### Spectator Services and Life Satisfaction

Life satisfaction is an individual’s cognitive judgment of the quality of life and is one of the most basic indicators of subjective well-being (Rice, 1984; Diener et al., 1999). Participation in sports and engagement in physical activities improve life satisfaction by fulfilling people’s basic psychological needs such as competence, autonomy, and relatedness, which in turn promote life satisfaction (Ryan and Deci, 2001; Funk et al., 2011a; Sato et al., 2016b). Similarly, recent studies have demonstrated that good sport event experiences may have positive effects on not only spectators’ behavioral responses but also their overall assessment of physical and mental health (Funk et al., 2011a; Sato et al., 2014, 2017; Du et al., 2015; Inoue et al., 2015, 2017; Baker et al., 2018; Hyun and Jordan, 2020). In other words, attendance at sport events might provide a context where people could obtain psychological benefits such as enjoyment and self-esteem and support personal development and learning, which in turn contribute to people’s life satisfaction (Iwasaki, 2007; Inoue et al., 2017).

Although life satisfaction may be attained through other behavioral means, live spectatorship represents a unique type of behavioral engagement that is especially the case during live spectating golf tournaments (Hansen and Gauthier, 1993, 1994; Lyu and Lee, 2013). Facilitated by core product services, walking on spectator paths with family or friends to enjoy the beautiful landscapes and scenery of golf courses is a common practice among golf spectators (Hansen and Gauthier, 1993, 1994; Lyu and Lee, 2013; Watanabe et al., 2013; Watanabe and Zhang, 2019). Perhaps most importantly, by closely observing sporting excellence and actively learning from professional golfers, spectators could have the opportunity to achieve personal development, which is critical for making one’s life meaningful and satisfying (Iwasaki, 2007). Peripheral services such as foodservice, shows, concerts, and staff courtesy at a tournament are inherently associated with leisure activities that may help spectators gain positive emotions, which in turn enhance and maintain their life satisfaction (Byon et al., 2013). In brief, given the meaning derived from and the needs fulfilled by the level of physical activity associated with golf spectatorship, spectators might possess a positive evaluation of life (Iwasaki, 2007; Inoue et al., 2017). Consistent with the social-ecological perspective on the influence of social and physical environments on population health, it can therefore be argued that golf is a unique spectator sport with salient participation and socialization elements enabled by spectator services that assimilate positive psychological resources into spectating (Inoue et al., 2017). As such, the current study tested the following hypotheses.

H5: Core product services would positively correlate with life satisfaction.
H6: Peripheral services would positively correlate with life satisfaction.

#### Length of Stay, Physical Activity, Self-Rated Health, and Life Satisfaction

Self-rated health is one’s perception of one’s own health and has been commonly measured in epidemiological and gerontological research (e.g., Mossey and Shapiro, 1982; Helliwell, 2003; Huang and Humphreys, 2012). It has been found to have a strong correlation with life satisfaction (Helliwell, 2003; Huang and Humphreys, 2012). Helliwell (2003) noted that self-reported health was one of the constructs that explained most variance in life satisfaction. Huang and Humphreys (2012) found that a supportive sport environment was positively associated with individuals’ self-reported health. More recently, Inoue et al. (2018) discovered that sporting event attendance was positively correlated with self-rated health over a 12-year period. Their findings provided support for the notion that sport spectatorship is an important leisure activity that might generate positive perceptions of general health and contribute to positive health outcomes. In this sense, golf tournaments may have greater health impact on spectators because golf spectatorship involves walking as a form of physical activity. Researchers have suggested that a lot of golf spectators attend golf events with the purpose to exercise or participate in physical activity so that they could obtain potential health benefits (Hansen and Gauthier, 1993, 1994; Robinson and Carpenter, 2002; Robinson et al., 2004; Lyu and Lee, 2013; Watanabe et al., 2013). Spectators may have the opportunity to acquire multiple physical and physiological benefits including improvement of cardiovascular and respiratory capacity, muscle strength, weight loss, and escape from physical stressors while walking on golf courses and learning diverse golf skills through spectating professional golf players’ performances (Hansen and Gauthier, 1993, 1994; Lyu and Lee, 2013; Watanabe et al., 2013). Murray et al. (2017b) revealed that many spectators attended golf events to obtain HEPA, while the rest still gained incidental HEPA through observing particular golfers or courses. Murray et al. (2019) suggested that when golf spectators were informed about the potential health benefits of golf spectatorship, they showed greater interest in participating in and spectating golf. Sato et al. (2016a) stated that the pleasant aspect of physical activity is likely to bring about good feelings in life, which might be conducive to self-rated health and life satisfaction. To sum up, higher step counts may be associated with higher levels of health perception and life satisfaction as spectators with higher step counts are more conscious about their health outcomes. As such, the following hypotheses were developed for the current study.

H7: Length of stay would positively correlate with step counts during the tournament.
H8: Step counts during the tournament would positively correlate with self-rated health.
H9: Step counts during the tournament would positively correlate with life satisfaction.
H10: Self-rated health would positively correlate with life satisfaction.

## Materials and Methods

### Participants

An on-site survey was conducted at the 2018 Bridgestone Open of the regular tour tournament in Japan, which was organized and operated by the Bridgestone Corporation. This tournament was a professional golf tournament held on October 18–21 at the Sodegaura Country Club’s Sodegaura Course in Sodegaura City. The current study was approved by the ethics committee of the Hiroshima University of Economics and the tournament organizer. The questionnaire had a cover letter that provided information on involved institutions, ethical guidelines, and data protection. Participants must sign a consent form before proceeding with the survey. Of the respondents, 64.3% were male and 35.7% were female. Approximately 44% of the respondents were at least 60 years of age, 30% were 50–59 years old, and 19% were 40–49 years old. A vast majority of spectators were golf players (78.5%). Among the spectators, 19.9% stayed at the event for less than 4 h, 42.8% for 4–6 h, and 37.3% stayed more than 6 h.

### Instrumentation

A questionnaire was formulated to measure spectators’ perceived core product services, perceived peripheral services, length of stay, step counts, self-rated health, and life satisfaction. The questionnaire was designed based on Hinkin’s (1998) guidelines of (a) formulating preliminary measures via procedures including a comprehensive review of related literature, on-site observations of previous golf events, and dialogues with a group of event marketers and event attendees of various golf tournaments, (b) conducting a test of content validity through a panel of experts, and (c) conducting a confirmatory factor analysis (CFA). For sample description purpose, variables for demographic information were also included in the questionnaire.

To measure the tournament’s core product services and peripheral services, measurement scales developed in a few critical studies were taken into consideration (e.g., Hansen and Gauthier, 1993, 1994; Wakefield and Sloan, 1995; Watanabe and Zhang, 2019). As a result, 10 items were developed to measure core product services and 17 items were developed to measure peripheral services. Five items assessing life satisfaction were derived from Sato et al. (2014, 2016b) work. The aforementioned items were slightly modified and adapted to fit with the golf tour tournament setting in Japan and were measured in a 6-point Likert-type scale ranging from 1 (*strongly disagree*) to 6 (*strongly agree*). Self-rated health was assessed by a single item asking respondents to rate their current state of general health on a 6-point scale ranging from 1 (*very unhealthy*) to 6 (*very healthy*) (Helliwell, 2003). Finally, step counts were measured in numerical values by spectators who utilized personal pedometers or smartphone pedometers to decide on the number of steps from time of entry to the venue until exit (see Appendix).

### Procedures

Permission to conduct the survey was obtained from the event management prior to the tournament. The paper-pencil questionnaires were distributed in the afternoon hours to spectators at food court areas and the exit gate. Trained students majoring in sport management monitored tournament progress, approached spectators resting in a food court area or heading to the exit gate, and helped with the survey administration. Spectators were asked to answer the following questions: “Are you going home” (yes); “did you record step counts during the tournament using a pedometer or a cellphone app” (yes); and “how many steps did you walk in this tournament” (number of steps)? Of the 600 questionnaires distributed during the 4-day tournament, 554 questionnaires were retrieved with a return rate of 92.3%. However, in this study we only included spectators who responded to the questions with the answers in the parentheses. This led to a total of 306 usable observations. The demographic characteristics of our sample were largely consistent with those of general golf spectators in Japan. According to past research, approximately 65–70% of spectators were male, 40% were 60 years of age or older, 25% were 50–59 years old, and 20% were 40–49 years old (Watanabe et al., 2013; Bridgestone Sports Co., Ltd, 2017; The Golf Tournament Promotion Association of Japan, 2018; Watanabe and Zhang, 2019). In addition, to control self-selection bias, we examined whether there were demographic and event-specific differences between participants who measured step counts and those who did not. No difference was found in terms of gender (χ^2^ = 0.53, df = 1, *p* = 0.48), age (χ^2^ = 28.81, df = 4, *p* = 0.14), golf rounds (χ^2^ = 3.75, df = 3, *p* = 0.29), companion (χ^2^ = 10.16, df = 6, *p* = 0.12), and length of stay (χ^2^ = 1.77, df = 2, *p* = 0.41), respectively.

### Data Analyses

Statistical analyses were performed using procedures in IBM SPSS Statistic 22.0 and IBM SPSS Amos 22.0. First, descriptive statistics (means, standard deviations, correlations, and checks for normality) were computed for variables using IBM SPSS Statistic 22.0. Second, confirmatory factor analyses (CFA) were conducted for the measurement model. Then, structural equation modeling (SEM) analyses examining direct and indirect effects in the proposed conceptual model were performed by using procedures in IBM SPSS Amos 22.0 with the maximum likelihood (ML) estimation. The measurement property of the structural model was first examined, followed by an assessment of the proposed structural relationships (Anderson and Gerbing, 1988). Goodness of fit indices were assessed by using the comparative fit index (CFI), normed chi-square (*χ^2^/df*), standardized root mean square residual (SRMR), and root mean square error of approximation (RMSEA). The reliability of the measures was assessed by Cronbach’s alpha, Average Variance Extracted (AVE), and composite reliability. The convergent validity and discriminant validity were examined by factor loadings and the inter-factor correlations (Fornell and Larcker, 1981; Kline, 2005; Hair et al., 2010).

## Results

Descriptive statistics for the variables are presented in Table 1. We started to assess the normality of dataset at the univariate level with skewness and kurtosis values. As shown in the table, all items had skewness value ranging from −2.80 to 1.03 and kurtosis value ranging from −0.92 to 2.64. Specifically, only one item’s skewness and kurtosis values were slightly above the suggested value of ±2.58 (Ghasemi and Zahediasl, 2012), suggesting that our data did not substantially deviate from normality. Further, the Mardia (1970) test was conducted to assess the multivariate normality of data distribution. As the results, the current data deviated from normality only at the multivariate level (Mardia’s skewness statistics = 221.92, *p* < 0.001; Mardia’s kurtosis statistics = 1307.98, *p* < 0.001). Therefore, the ML estimation method was used for the CFA because it was robust even when applying to some conditions non-normally distributed data (Hoyle and Panter, 1995; Olsson et al., 2000).

**TABLE 1:** Descriptive statistics for variables included in the study.

	**Variables**	**Mean**	**SD**	**Skewness**	**Kurtosis**
Core product services	Presence of famous players	4.65	1.48	−1.38	1.72
	Showing of favorite players	4.85	1.51	−1.62	2.17
	Being able to follow players	3.73	1.70	−0.59	−0.37
	Opportunity to watch players closely	5.41	0.86	−1.96	2.07
	Being able to watch players’ practice closely	4.75	1.40	−1.17	1.03
	Players’ high performance	5.18	1.01	−1.20	0.87
	Opportunity to learn from players	4.16	1.70	−0.78	−0.19
	Ease of walking on golf course	4.37	1.47	−0.84	0.20
	Enjoyable scenery of golf course	4.46	1.31	−1.09	1.42
	Pleasant atmosphere of the event	4.38	1.39	−0.85	0.49
Peripheral service*s*	Foodservice quality	4.12	1.14	−0.22	−0.09
	Price of foodservice	3.58	1.25	−0.09	−0.13
	Variety of golf shop offerings (e.g., souvenirs, equipment)	3.55	1.21	−0.31	0.88
	Ancillary activities (e.g., shows, concerts, autograph session)	3.63	1.28	−0.31	0.83
	Ticket promotion	3.82	1.41	−0.45	0.17
	Convenience of buying ticket	4.04	1.45	−0.63	0.41
	Event information accessibility	4.01	1.38	−2.80	2.64
	Crowd control	4.47	1.06	−0.46	0.29
	Spectating area accessibility	4.42	1.05	−0.32	−0.30
	Spectating area convenience	4.28	1.08	−0.30	−0.32
	Scoreboard quality	4.67	0.99	−0.40	−0.61
	Staff courtesy	4.55	1.05	−0.28	−0.55
	Restroom cleanliness	4.03	1.24	−0.45	0.06
	Parking accessibility	4.26	1.22	−0.40	−0.11
	Parking availability	3.07	1.90	−0.38	−0.88
	Parking cost	2.73	1.80	−0.15	−0.92
	Public transportation accessibility	4.49	1.46	−1.28	1.68
Life satisfaction	I am satisfied with my life	4.49	1.15	−0.63	0.44
	If I could live my life over, I would change almost nothing	4.45	1.14	−0.55	0.25
	In most ways my life is close to ideal	4.54	1.13	−0.47	−0.25
	The conditions in my life are excellent	4.78	1.03	−0.67	0.54
	So far, I have gotten the important things I want in life	4.53	1.10	−0.37	−0.27
	*Length of stay (in hours)*	5.05	1.60	0.36	0.14
	*How many steps did you walk in this tournament?*	8,714.35	3658.06	0.63	1.02
	*Self- rated health*	4.85	0.86	−0.91	1.27

Subsequently, CFAs were proceeded. To reduce the complexity of tested relationship model and improve the reliability of research results, both core services and peripheral services were treated as second-order factors. As shown in Table 2, factor loadings for items were all greater than 0.60. Fit indices of second-order core product services were acceptable: χ^2^/*df* = 3.07, CFI = 0.92, RMSEA = 0.08 with 90% CI = 0.06–0.09, SRMR = 0.06 (Bollen, 1989; Kline, 2005). Similarly, fit indices of second-order peripheral services were satisfactory: χ^2^/*df* = 2.73, CFI = 0.93, RMSEA = 0.05 with 90% CI = 0.06–0.08, SRMR = 0.05. As to the overall measurement, its fit indices were *χ^2^/df* = 1.70, CFI = 0.93, RMSEA = 0.04 with 90% CI = 0.04–0.05, SRMR = 0.05. In addition, validity and reliability values for latent factors were also above the recommended threshold. As shown in Table 2, the values of Cronbach’s alpha (ranging from 0.71 to 0.92), AVE (ranging from 0.54 to 0.75), and composite reliability (ranging from 0.82 to 0.93) were all above the suggested values (α ≧ 0.70, CR ≧ 0.60 by Bagozzi and Yi, 1988; AVE ≧ 0.50 by Fornell and Larcker, 1981). Factor loadings were larger than or close to 0.70, indicating good convergent validity (Hair et al., 2010). Inter-factor correlations were below the cutoff value of 0.85, ranging from −0.01 to 0.61 (Table 3), confirming good discriminant validity of measurement model (Hair et al., 2010). We also assessed whether there was a threat of common method bias (CMV) by using Harman’s single factor test. The result showed that no single factor could explain more than 50% of the variance, indicating that CMV was not a serious issue in our study.

**TABLE 2:** Indicator loadings, critical ratios, construct reliability, average variance extracted for the measurement model.

**Factors**	**Items**	**Loadings**	**α**	**CR**	**AVE**
Core product services	*Player attraction*	*0.77*	0.71	0.82	0.60
	Presence of famous players	0.84			
	Showing of favorite players	0.79			
	Being able to follow players	0.69			
	*Event attractiveness*	*0.72*	0.75	0.83	0.54
	Opportunity to watch players closely	0.78			
	Being able to watch players’ practice closely	0.77			
	Players’ high performance	0.70			
	Opportunity to learn from players	0.70			
	*Course characteristics*	*0.74*	0.86	0.90	0.75
	Ease of walking on golf course	0.90			
	Enjoyable scenery of golf course	0.89			
	Pleasant atmosphere of the event	0.81			
	First-order factor model: CMIN/DF = 3.07, CFI = 0.92, SRMR= 0.06, RMSEA = 0.08 (90% CI = 0.06–0.09)				
	Second-order factor model: CMIN/DF = 3.06, CFI = 0.92, SRMR= 0.06, RMSEA = 0.08 (90% CI = 0.06–0.09)				
Peripheral services	*Event services*	*0.72*	0.85	0.89	0.67
	Foodservice quality	0.82			
	Price of foodservice	0.82			
	Variety of golf shop offerings (e.g., souvenirs, equipment)	0.82			
	Ancillary activities (e.g., shows, concerts, autograph session)	0.81			
	*Event information*	*0.79*	0.84	0.88	0.71
	Ticket promotion	0.92			
	Convenience of buying ticket	0.88			
	Event information accessibility	0.71			
	*Event amenity*	*0.79*	0.91	0.93	0.69
	Crowd control	0.91			
	Spectating area accessibility	0.89			
	Spectating area convenience	0.84			
	Scoreboard quality	0.82			
	Staff courtesy	0.81			
	Restroom cleanliness	0.69			
	*Parking and transportation*	*0.76*	0.81	0.89	0.68
	Parking accessibility	0.71			
	Parking availability	0.93			
	Parking cost	0.92			
	Public transportation accessibility	0.70			
	First-order factor model: CMIN/DF = 2.73, CFI = 0.93, SRMR= 0.05, RMSEA = 0.05 (90% CI = 0.06–0.08)				
	Second-order factor model: CMIN/DF = 2.76, CFI = 0.93, SRMR= 0.05, RMSEA = 0.05 (90% CI = 0.06–0.08)				
Life satisfaction	I am satisfied with my life	0.89	0.92	0.92	0.69
	If I could live my life over, I would change almost nothing	0.89			
	In most ways my life is close to ideal	0.86			
	The conditions in my life are excellent	0.78			
	So far, I have gotten the important things I want in life	0.72			

*Cronbach’s alpha (α), construct reliability, (CR), averaged variance extracted (AVE).*

**TABLE 3:** Inter-concept correlations among variables.

			**1**	**2**	**3**	**4**	**5**	**6**	**7**	**8**	**9**	**10**	**11**
1	Player attraction	Pearson	–										
		Kendall											
		Spearman											
2	Event attractiveness	Pearson	0.33***	–									
		Kendall	0.25***										
		Spearman	0.32***										
3	Course characteristics	Pearson	0.30***	0.31***	–								
		Kendall	0.24***	0.25***									
		Spearman	0.32***	0.32***									
4	Event services	Pearson	0.33***	0.39***	0.36***	–							
		Kendall	0.24***	0.28***	0.25***								
		Spearman	0.32***	0.37***	0.33***								
5	Event information	Pearson	0.29***	0.36***	0.27***	0.47***	–						
		Kendall	0.22***	0.23***	0.23***	0.35***							
		Spearman	0.29***	0.30***	0.30***	0.46***							
6	Event amenity	Pearson	0.25***	0.26***	0.30***	0.47***	0.47***	–					
		Kendall	0.21***	0.23***	0.26***	0.35***	0.37***						
		Spearman	0.28***	0.30***	0.34***	0.46***	0.48***						
7	Parking and transportation	Pearson	0.28***	0.30***	0.25***	0.40***	0.39***	0.43***	–				
		Kendall	0.18***	0.19***	0.16***	0.26***	0.27***	0.32***					
		Spearman	0.24***	0.26***	0.22***	0.35***	0.36***	0.43***					
8	Life satisfaction	Pearson	0.33***	0.42***	0.32***	0.31***	0.35***	0.34***	0.24***	–			
		Kendall	0.24***	0.31***	0.24***	0.23***	0.25***	0.27***	0.15***				
		Spearman	0.32***	0.41***	0.33***	0.30***	0.33***	0.35***	0.21***				
9	Length of stay (in hours)	Pearson	0.11*	0.10	0.09	0.06	0.18**	0.07	0.03	0.21***	–		
		Kendall	0.10*	0.06	0.04	0.03	0.14**	0.04	0.01	0.16***			
		Spearman	0.13*	0.07	0.05	0.04	0.18**	0.06	0.01	0.21***			
10	Step counts	Pearson	0.07	0.10*	0.09	−0.02	0.04	0.02	0.03	0.02	0.19***	–	
		Kendall	0.06	0.10*	0.06	0.00	0.01	0.01	0.01	0.02	0.15***		
		Spearman	0.09	0.14*	0.09	0.00	0.01	0.01	0.01	0.03	0.20***		
11	Self-rated health	Pearson	0.05	0.21***	0.15**	0.16**	0.08	0.17**	0.16**	0.21***	0.03	0.11	–
		Kendall	0.07	0.18***	0.13**	0.14**	0.07	0.12**	0.12**	0.17***	0.04	0.07	
		Spearman	0.08	0.23***	0.17***	0.18***	0.09	0.15**	0.15**	0.21***	0.04	0.09	
Mean			4.41	4.88	4.41	3.72	3.96	4.40	3.64	4.56	5.05	8714.36	4.85
SD			1.17	0.90	1.23	1.01	1.16	0.89	1.11	0.93	1.60	3658.06	0.86

* *p* < 0.05, ** *p* < 0.01, *** *p* < 0.001.

**FIGURE 2:**
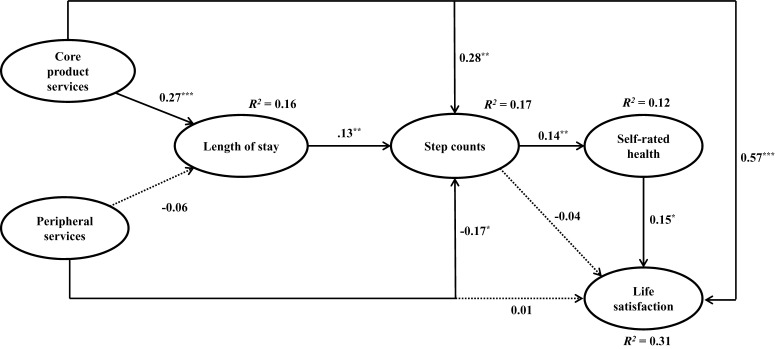
Resulted model derived from structural equation model analyses. Solid lines indicate significant paths; Dotted lines indicate insignificant paths. *p < 0.05, **p < 0.01, ***p < 0.001.

Research hypotheses were tested by conducting SEM analyses. Fit indices of relationship model were above average: *χ^2^/df* = 2.25, CFI = 0.96, RMSEA = 0.05 with 90% CI = 0.04–0.06, SRMR = 0.06. As shown in Table 4 and Figure 2, core product services positively correlated with spectator’s length of stay at the tournament (β = 0.27, *p* < 0.001), confirming H1. However, peripheral services did not have a significant effect on length of stay, rejecting H2. Core product services positively (β = 0.28, *p* < 0.01) and peripheral services negatively (β = −0.17, *p* < 0.05) correlated with step counts, supporting H3 but rejecting H4. As to life satisfaction, only core product services positively associated with it (β = 0.57, *p* < 0.001), supporting H5 and rejecting H6. Further, length of stay positively correlated with step counts (β = 0.13, *p* < 0.01), supporting H7; step counts positively associated with self-rated health (β = 0.14, *p* < 0.01), which further positively linked to life satisfaction (β = 0.15, *p* < 0.05), supporting H8 and H10. The direct effect of step counts on life satisfaction was not supported, rejecting H9. Albeit minimal, the indirect effect of step counts on life satisfaction via self-related health was significant (β = 0.02, *p* < 0.01), suggesting a full mediating effect. Overall, the structural model explained 16% of the length of stay, 17% of step counts, 12% of self-rated health, and 31% of life satisfaction.

**TABLE 4:** Standardized coefficients of model paths.

**Effects**			**β**	***t***	**SE**	**Hypotheses**	
Core product services	⇒	Length of stay (in hours)	0.27***	3.46	0.16	H1	Supported
	⇒	Step counts	0.28**	2.59	0.21	H3	Supported
	⇒	Life satisfaction	0.57***	5.42	0.11	H5	Supported
Peripheral services	⇒	Length of stay (in hours)	–0.06	–0.81	0.14	H2	Not supported
	⇒	Step counts	−0.17*	–2.08	0.18	H4	Not supported
	⇒	Life satisfaction	0.01	–0.12	0.09	H6	Not supported
Length of stay (in hours)	⇒	Step counts	0.13**	2.62	0.06	H7	Supported
Step counts	⇒	Self-rated health	0.14**	2.60	0.05	H8	Supported
Step counts	⇒	Life satisfaction	–0.04	–0.71	0.03	H9	Not supported
Self-rated health	⇒	Life satisfaction	0.15*	1.98	0.02	H10	Supported

		*R*^2^	Fit indices				

Length of stay (in hours)		0.16	CMIN/DF = 2.25, CFI = 0.96 RMSEA = 0.05 (90%CI=0.04–0.06), SRMR = 0.06				
Step counts		0.17					
Self-rated health		0.12					
Life satisfaction		0.31					

* *p* < 0.05, ** *p* < 0.01, *** *p* < 0.001.

## Discussion

The findings of this study help clarify spectators’ consumption and health-rated behaviors by examining the antecedents and consequences of event-related factors at a professional golf tournament in Japan. Intuitively, the tangible and intangible factors that are related to the core product services and peripheral spectator services of a golf tournament are often viewed as being important preconditions for attracting spectators and influencing their consumption outcomes. Previous studies have revealed that the core product services and peripheral services of sporting events have an impact on spectators’ desires to stay, their event satisfaction levels, and their repatronage intentions (Tsuji et al., 2007; Byon et al., 2013; Watanabe et al., 2018). Nevertheless, it was further seen in this study that the tournament-related spectator services would have an influence on physical activity, which could in turn influence self-rated health, and subsequently impact life satisfaction. Inoue et al. (2017) stated that the significant relationship between spectator attendance and life satisfaction highlights the unique ability of behavioral engagement with spectator sporting events to predict life satisfaction. Emphasizing the sense of accomplishment fulfilled in sport spectatorship is recommended for the well-being of the spectators (Kim and James, 2019). In line with the social-ecological perspective (Stokols, 1996), the findings of the current study provide support for the potential correlation between sport event-related and health-related variables. Concerning the core product services, in particular, it makes reasonable sense that seeing highly skilled golfers compete in person positively affects spectators’ length of stay and step counts at the tournament (Hansen and Gauthier, 1993, 1994; Watanabe et al., 2013). Past research has shown that the positive performance of star soccer players can have a positive effect on their fans’ physical activity (Mutter and Pawlowski, 2014), and this effect appears to carry over to golf. Events featuring Tiger Woods and other superstars, for example, tend to receive the most attention and the most massive followings (Keefer, 2018). As fans follow these famous golfers around, they are more likely to achieve higher step counts and tend to gain higher levels of life satisfaction. Golf events could encourage players to interact more frequently with the fans. In this way, a vital element of the core product can be linked more directly to spectator health and life satisfaction. The tournament observed in this study was also played on a reputable course that had a history and tradition associated with it; thus, this core factor, too, may have further motivated spectators to attend the event and remain active at the event as they were inspired by the competitive nature of the tournament and its associated aesthetics. Tudor-Locke and Bassett (2004) recommended as part of a general physical activity plan that people accumulate 10,000 steps each day. Walking 18 holes in a standard round of golf certainly meets the recommendation to accumulate 10,000 steps per day, as would following a golfer or series of golfers around an expansive course for several hours (Kobriger et al., 2006). In other words, attending a golf tournament can almost be as beneficial to one’s health as playing the game itself (Murray et al., 2017a, b). Spectators who have more steps may increase their self-rated health through spectating golf events.

Based on these suggestions, one’s ability to remain close to the golfer and observe his or her mechanics and demeanor may provide more utility than pure entertainment value. Moreover, according to Funk and James (2001, 2006), a consumer characterized by high allegiance provides repeat consumption (i.e., continued participation in an activity) based on the symbolic value of the activity, the amount of relevant knowledge, and the positive emotional response. Thus, when a spectator already has an allegiance to golf, they may want to enable further exploration of this meaning through a process such as spectatorship that links golf and life satisfaction. This is supported by the positive effect of core product services on life satisfaction (β = 0.57, *p* < 0.001).

Shifting to the influence of peripheral services on the spectators, the only significant effect was a small, negative link between peripheral services and step counts (β = −0.17, *p* < 0.05). Though the small effect size limits the need for extensive discussion, the negative relationship makes sense given that many peripheral services (e.g., parking accessibility, special seating areas, merchandise stores, and concession areas) are designed for convenience. Convenience, by nature, will limit step counts and keep people from exerting too much energy. If tournament organizers wanted to mitigate this adverse effect, they could place kiosks and concession areas further back in the course, limit seating in these areas, and ensure that most of the focus remains on the core product of following golfers around the course. As many researchers (Wakefield and Sloan, 1995; Wakefield et al., 1996; Zhang et al., 2005; Mullin et al., 2014) have stated, the peripheral services are supplementary to the process of providing the core product. Spectators attending a sporting event are likely attending to experience the core product (i.e., the competition itself); peripheral services, as the name implies, are secondary to the main event. Therefore, it is not too surprising that peripheral services are incapable of keeping people longer. Most likely, the core product will have to do that. Having seen that peripheral services have a negative effect on step counts, it may actually be good for spectator health if they are not influential in keeping patrons at the event. There was also no significant relationship between peripheral services and life satisfaction; again, this is likely explained by the reality that consumers are paying for, and basing their expectations on, the core product. Their experiences with the core product are the ones capable of significantly influencing their life satisfaction, as was seen in the Figure 2 model (β = 0.57, *p* < 0.001).

## Practical Implications

As noted in parts of the opening discussion, some of these results can be practically applied to areas of sport event management and marketing. The primary application stems from one of the unique attributes of golf spectatorship in that it is rooted in physical activity and consists of patrons who play the game even at an old age. Indeed, golf sets itself up as a rare form of spectator sport where spectators – in an effort to follow star players around the course or see the intricacies of a course’s different holes – generally remain on the move. The health benefits generated while observing a competition are bolstered when they attend these events and witness the performances of the players firsthand. In this sense, spectating golf carries with it a variety of health benefits (Parkkari et al., 2000; Kobriger et al., 2006; Murray et al., 2017a, 2019).

Marketers and planners of similar events should incorporate these unique health-related benefits into their plans and promotional efforts by advertising and incentivizing them more frequently. Perhaps more people would attend if they were more aware of the associated health benefits and how a physically active lifestyle can lead to higher levels of satisfaction in life. Health-related sponsors could also be recruited to further facilitate this unique relationship and bring in additional funding to the event. Furthermore, if it is the core elements of an event that give patrons a desire to remain at the course for a longer duration, these elements should be logically emphasized. Increased accessibility to star players, such as what is seen at Augusta National during the practice rounds for the Masters, will likely lead to increased physical activity and life satisfaction for those in attendance. Similarly, a course layout that features unique holes will encourage spectators to avoid sitting in one location throughout the event, as would a level of competition that is close and unpredictable. This last feature certainly presents a unique challenge for golf event marketers who cannot control the performances of the key players. Even so, such situations may be mitigated by the sheer presence of a popular, skilled golfer who can still attract large followings. Attracting and raising awareness for these golfers is, therefore, an important task. In being close to the professional players and seeing their skills and abilities firsthand, patrons are also likely to leave with knowledge of new swing mechanics or techniques that they could try on their own. The PGA Tour’s slogan for many years, after all, was “These guys are good” (Beall, 2018); perhaps some consumers want to replicate this in their own right. Allowing spectators to view the players while they practice and warm up is a helpful way in which this activity could be facilitated.

Nevertheless, it is important to mention the peripheral services, the lack of impact they appear to have on duration of stay and life satisfaction, and the negative impact they have on step counts at the event. In regards to the latter, concession stands, merchandise shops, customer service attendants, and other specialized areas could be placed throughout the course to encourage walking and reward those who are being physically active. Rather than having everything at the beginning of the course or in the clubhouse area, certain locations could be strategically placed so as to motivate people to walk there and satisfy those who do. For example, if spectators achieve a set number of steps, they could have a photograph taken with a player. In this way, patrons will be happier and required to stay longer – two things that benefit both the spectator and those running the event.

Lastly, both of the core and peripheral features and their links to physical activity and satisfaction could be enhanced by merging gamification elements with the spectator experience. Gamification, a term frequently used to refer to the implementation of game designs in non-game contexts (Koivisto and Hamari, 2019), is being paired with technology to direct people’s motivations toward intrinsically motivated experiences and behaviors. By allowing spectators to check-in via mobile apps and other technologies, track steps, and earn badges or other rewards for reaching certain locations or physical milestones on the course, spectators could become more involved in a process that suits both their fandom and physical activity. Though it might go against the grains of tradition to allow mobile devices at golf events, there have been recent movements to make the game more laid-back and enjoyable.

In 2017, the PGA and European tours announced that they were lifting the bans on mobile phones that had been in place at professional golf tournaments for years (Berhow, 2017). “The softening of the mobile device policy is a step to make the Tour more fan-friendly,” said one PGA Tour spokesperson. “Fans will also be allowed to share their tournament photos and videos via social media” (Berhow, 2017, para. 4). Ultimately, these loosened restrictions on the use of mobile devices and other social technologies should make golf tournaments a more conducive setting for those who want a more social and interactive environment. Event organizers might also find that these relaxed rules promote healthier habits and a stronger connection to the sport as spectators will now be motivated to walk around and take pictures of their favorite golfers at different hole locations. Therefore, tournament policies and plans should be seamlessly merged with creative applications and technologies that can enhance the spectator experience through physical activity and intrinsic rewards. In this way, event organizers and promoters can emphasize the core product while encouraging healthier habits among their spectators, healthier habits that may lead to more satisfied consumers.

## Limitations and Future Directions

This study has several limitations, the first of which lies in the design of cross-sectional survey in which participants’ responses were subjected to short-term situational factors (e.g., personal mood, atmosphere, and specific needs during the survey period). This, to some extent, reduced the reliability and validity of research findings. Future studies are strongly recommended to employ a longitudinal design and collect panel data to better decipher golf spectators’ health-related behaviors. Another limitation of the study involves the generalizability of the findings. While the sound characteristics of its measures would allow it to be applied to similar research contexts in the future, this questionnaire was developed specifically for the study of a Japanese professional golf event. It should be noted that our sample could be susceptible to potential sampling bias that would influence the generalizability of the study. Future research is advised to adopt a more effective sampling method to tackle this issue. Further, diverse spectator roles, purposes, and segmentations according to various background variables (e.g., gender, age, ticket type, day of the week) should be included and integrated more thoroughly in future studies. Seeing as cost and affordability may play a key role in the sports one watches or participates in, ticket type and its corollary of consumer income might be particularly important for future analyses to consider. Golf in particular is often viewed as being a “higher-class” sport (An and Sage, 1992), so one could see if personal income has an impact on the likelihood that a consumer will consistently play the sport after attending an event. A spectator’s involvement with peripheral elements of the event and their overall satisfaction may also be influenced by this variable, further highlighting the need for future studies to incorporate it in their analyses.

When considering health-related variables, step counts, the physical activity measure in our study, may have a high variability depending on the characteristics of a course (Kobriger et al., 2006). Robinson and Carpenter (2002) highlighted how certain characteristics such as course layout and proximity to the players differ among spectators, meaning it may be necessary to include these characteristics in future studies in an effort to explore potential moderating effects. In a similar sense, it might be interesting to include variables related to the “competitive balance” of the tournament. Closer competitions in which two or more players are battling it out will likely draw bigger crowds, leading to more vigorous walking (exercise) as patrons follow the action instead of remaining stationary and attempt to beat the crowds to prime viewing locations. The significantly positive influence of the core elements of consumer demand (which would include the players and their performances) on patrons’ desires to stay at the event may already be capturing this effect in-part, but variables that more accurately capture the effects of a close match on exercise vigor and satisfaction could prove more insightful. Lastly, as prior research has noted (e.g., Sato et al., 2016a), actual behavior may be inconsistent with self-reported measures, and focusing on actual behavior as the outcome variable may yield more accurate results (Sato et al., 2015). Therefore, the gap between self-reported and actual behavioral measurement could be melded in future studies.

## Data Availability Statement

All datasets generated for this study are included in the article/supplementary material.

## Ethics Statement

The current study was approved by the Ethics Committee of the Hiroshima University of Economics and the tournament organizer. The participants provided their written informed consent to participate in this study.

## Author Contributions

YW, TQ, and JZ contributed to the conception and design of the study. YW collected the data and analyzed the data. YW and TQ wrote the draft of the manuscript. JW, NP, and JZ contributed to the manuscript revision. All authors contributed to the article and approved the submitted version.

## Conflict of Interest

The authors declare that the research was conducted in the absence of any commercial or financial relationships that could be construed as a potential conflict of interest.

## References

[B1] Aizawa K. Wu J. Inoue Y. Sato M. (2018). Long-term impact of the Tokyo 1964 Olympic Games on sport participation: a cohort analysis. *Sport Manag. Rev.* 21 86–97. 10.1016/j.smr.2017.05.001

[B2] An M. Sage G. H. (1992). The golf boom in south korea: serving hegemonic interests. *Sociol. Sport J.* 9 372–384.

[B3] Anderson D. R. Gerbing D. W. (1988). Structural equation modeling in practice: a review and recommended two-step approach. *Psychol. Bullet.* 103 411–423. 10.1037/0033-2909.103.3.411

[B4] Athanassopoulos A. Gounaris S. Stathakopoulos V. (2001). Behavioural responses to customer satisfaction: an empirical study. *Eur. J. Market.* 35 687–707. 10.1108/03090560110388169

[B5] Bagozzi R. P. Yi Y. (1988). On the evaluation of structural equation models. *J. Acad. Market. Sci.* 16 74–94.

[B6] Baker B. J. Jordan J. S. Funk D. C. (2018). Run again another day: the role of consumer characteristics and satisfaction in repeat consumption of a sport-related experience product. *J. Sport Manag.* 32 38–52. 10.1123/jsm.2017-0042

[B7] Banyard P. Shevlin M. (2001). Responses of football fans to relegation of their team from the English Premier League: PTS? *Irish J. Psychol. Med.* 18 66–67. 10.1017/s0790966700006352 30440166

[B8] Bauman A. Ainsworth B. E. Sallis J. F. Hagströmer M. Craig C. L. Bull F. C. (2011). The descriptive epidemiology of sitting: a 20-country comparison using the International Physical Activity Questionnaire (IPAQ). *Am. J. Preven. Med.* 41 228–235. 10.1016/j.amepre.2011.05.003 21767731

[B9] Beall J. (2018). *Why you won’t Hear ‘These Guys Are Good’ From the PGA Tour Anymore. GolfDigest.com.* Available online at: http://www.golfdigest.com/story/why-you-wont-hear-these-guys-are-good-from-the-pga-tour-anymore (accessed April 30, 2019).

[B10] Berhow J. (2017). *PGA Tour Will Now Allow Spectators to Take Photos, Videos During Tournament Rounds. Golf.com.* Available online at: www.golf.com/tour-news/2017/08/24/pga-tour-adjusts-phone-policy-allows-spectator-photos-during-rounds (accessed April 30, 2019).

[B11] Bollen K. A. (1989). *Structural Equations With Latent Variables.* New York, NY: Wiley.

[B12] Braunstein J. R. Zhang J. J. Trail G. T. Gibson H. J. (2005). Dimensions of market demand associated with major league baseball spring training: development of a scale. *Sport Manag. Rev.* 8 271–296. 10.1016/s1441-3523(05)70042-5

[B13] Bridgestone Sports Co., Ltd (2017). *The Tour Tournament Spectator Research Data Book.* Tokyo: Bridgestone Sports Co., Ltd.

[B14] Brownson R. C. Eyler A. A. King A. C. Brown D. R. Shyu Y. L. Sallis J. F. (2000). Patterns and correlates of physical activity among US women 40 years and older. *Am. J. Public Health* 90 264–270. 10.2105/ajph.90.2.264 10667189 PMC1446154

[B15] Byon K. K. Zhang J. J. Baker T. A. (2013). Impact of core and peripheral services quality on consumption behavior of professional team sport spectators as mediated by perceived value. *Eur. Sport Manag. Q.* 13 232–263. 10.1080/16184742.2013.767278

[B16] Chalip L. (2006). Toward a distinctive sport management discipline. *J. Sport Manag.* 20 1–21. 10.1123/jsm.20.1.1

[B17] Chaudhury H. Campo M. Michael Y. Mahmood A. (2016). Neighborhood environment and physical activity in older adults. *Soc. Sci. Med.* 149 104–113. 26708246 10.1016/j.socscimed.2015.12.011 PMC10339378

[B18] Chelladurai P. (2014). *Managing Organizations for Sport and Physical Activity: A Systems Perspective*, 4th Edn New York, NY: Routledge.

[B19] Diener E. Suh E. M. Lucas R. E. Smith H. L. (1999). Subjective well-being: three decades of progress. *Psychol. Bullet.* 125 276–302. 10.1037/0033-2909.125.2.276

[B20] Du J. Jordan J. S. Funk D. C. (2015). Managing mass sport participation: adding a personal performance perspective to remodel antecedents and consequences of participant sport event satisfaction. *J. Sport Manag.* 29 688–704. 10.1123/jsm.2014-0225

[B21] Elling A. Van Hilvoorde I. Van Den Dool R. (2014). Creating or awakening national pride through sporting success: a longitudinal study on macro effects in the Netherlands. *Int. Rev. Soc. Sport* 49 129–151. 10.1177/1012690212455961

[B22] Fornell C. Larcker D. F. (1981). Evaluating structural equation models with unobservable variables and measurement error. *J. Market. Res.* 18 39–50. 10.1177/002224378101800104

[B23] Frawley S. Cush A. (2011). Major sport events and participation legacy: the case of the 2003 Rugby World Cup. *Managing leisure* 16 65–76. 10.1080/13606719.2011.532605

[B24] Funk D. C. Beaton A. Pritchard M. (2011a). The stage-based development of physically active leisure: a recreational golf context. *J. Leisure Res.* 43 268–289. 10.1080/00222216.2011.11950236

[B25] Funk D. Jordan J. Ridinger L. Kaplanidou K. (2011b). Capacity of mass participant sport events for the development of activity commitment and future exercise intention. *Leisure Sci.* 33 250–268. 10.1080/01490400.2011.564926

[B26] Funk D. C. James J. (2001). The psychological continuum model: a conceptual framework for understanding an individual’s psychological connection to sport. *Sport Manag. Rev.* 4 119–150. 10.1016/s1441-3523(01)70072-1

[B27] Funk D. C. James J. D. (2006). Consumer loyalty: The meaning of attachment in the development of sport team allegiance. *J. Sport Manag.* 20 189–217. 10.1123/jsm.20.2.189

[B28] Ghasemi A. Zahediasl S. (2012). Normality tests for statistical analysis: a guide for non-statisticians. *Int. J. Endocrinol. Metab.* 10 486–489. 10.5812/ijem.3505 23843808 PMC3693611

[B29] Giles-Corti B. Donovan R. J. (2002). The relative influence of individual, social and physical environment determinants of physical activity. *Soc. Sci. Med.* 54 1793–1812. 10.1016/s0277-9536(01)00150-2 12113436

[B30] Hair J. F. Black W. Babin B. J. Anderson R. E. (2010). *Multivariate Data Analysis*, 7th Edn Upper Saddle River, NJ: Pearson Prentice Hall.

[B31] Hansen H. Gauthier R. (1993). Spectators’ views of LPGA golf events. *Sport Market. Q.* 2 17–25.

[B32] Hansen H. Gauthier R. (1994). The professional golf product: Spectators’ views. *Sport Market. Q.* 3 9–16.

[B33] Helliwell J. F. (2003). How’s life? Combining individual and national variables to explain subjective well-being. *Economic Model.* 20 331–360. 10.1016/s0264-9993(02)00057-3

[B34] Hinkin T. R. (1998). A brief tutorial on the development of measures for use in survey questionnaires. *Organ. Res. Methods* 1 104–121. 10.1177/109442819800100106

[B35] Hoehner C. M. Ramirez L. K. B. Elliott M. B. Handy S. L. Brownson R. C. (2005). Perceived and objective environmental measures and physical activity among urban adults. *Am. J. Preven. Med.* 28 105–116. 10.1016/j.amepre.2004.10.023 15694518

[B36] Hoyle R. H. Panter A. T. (1995). “Writing about structural equation modeling,” in *Structural Equation Modeling: Concepts, Issues, and Applications*, ed. Hoyle R. H. (Thousand Oaks, CA: Sage), 158–176.

[B37] Huang H. Humphreys B. R. (2012). Sports participation and happiness: evidence from US microdata. *J. Econo. Psychol.* 33 776–793. 10.1016/j.joep.2012.02.007

[B38] Hyun M. Jordan J. S. (2020). Athletic goal achievement: a critical antecedent of event satisfaction, re-participation intention, and future exercise intention in participant sport events. *Sport Manag. Rev.* 23 256–270. 10.1016/j.smr.2019.01.007

[B39] Inoue S. Ohya Y. Tudor-Locke C. Tanaka S. Yoshiike N. Shimomitsu T. (2011). Time trends for step-determined physical activity among Japanese adults. *Med. Sci. Sports Exerc.* 43 1913–1919. 10.1249/mss.0b013e31821a5225 21448082

[B40] Inoue Y. Berg B. K. Chelladurai P. (2015). Spectator sport and population health: a scoping study. *J. Sport Manag.* 29 705–725. 10.1123/jsm.2014-0283

[B41] Inoue Y. Sato M. Filo K. Du J. Funk D. C. (2017). Sport spectatorship and life satisfaction: a multicountry investigation. *J. Sport Manag.* 31 419–432. 10.1123/jsm.2016-0295

[B42] Inoue Y. Sato M. Nakazawa M. (2018). Association between sporting event attendance and self-rated health: an analysis of multiyear cross-sectional national data in Japan. *Glob. Health Res. Policy* 3:13. 10.1186/s41256-018-0068-9 PMC5936628 29761159

[B43] Iwasaki Y. (2007). Leisure and quality of life in an international and multicultural context: What are major pathways linking leisure to quality of life? *Soc. Indicat. Res.* 82 233–264. 10.1007/s11205-006-9032-z

[B44] Japan Sports Agency (2018). *Second Sport Basic Plan.* Available online at: http://www.mext.go.jp/sports/en/b_menu/policy/sysbudget/secondsportbp.htm (accessed March 15, 2019).

[B45] Keefer Z. (2018). *The Tiger Effect Revs up PGA Championship Fans to a Frenzy. USAToday.com.* Available online at: https://www.usatoday.com/story/sports/golf/2018/08/11/tiger-woods-effect-revs-up-pga-championship-fans-frenzy/969508002/ (accessed April 10, 2019).

[B46] Kim J. James J. D. (2019). Sport and happiness: understanding the relations among sport consumption activities, long-and short-term subjective well-being, and psychological need fulfillment. *J. Sport Manag.* 33 119–132. 10.1123/jsm.2018-0071

[B47] Kim M. K. Zhang J. J. Ko Y. J. (2009). Dimensions of market demand associated with Taekwondo schools in North America: development of a scale. *Sport Manag. Rev.* 12 149–166. 10.1016/j.smr.2009.01.003

[B48] Kline R. B. (2005). *Principles and Practice of Structural Equation Modeling*, 2nd Edn New York, NY: Guilford.

[B49] Kobriger S. L. Smith J. Hollman J. H. Smith A. M. (2006). The contribution of golf to daily physical activity recommendations: how many steps does it take to complete a round of golf? *In Mayo Clin. Proc.* 81 1041–1043. 10.4065/81.8.1041 16901027

[B50] Koivisto J. Hamari J. (2019). The rise of motivational information systems: a review of gamification research. *Int. J. Inform. Manag.* 45 191–210. 10.1016/j.ijinfomgt.2018.10.013

[B51] Kras J. M. Larsen B. T. (2002). A comparison of the health benefits of walking and riding during a round of golf. *Int. Sports J.* 6 112–116.

[B52] Lam L. W. Chan K. W. Fong D. Lo F. (2011). Does the look matter? The impact of casino servicescape on gaming customer satisfaction, intention to revisit, and desire to stay. *Int. J. Hospit. Manag.* 30 558–567. 10.1016/j.ijhm.2010.10.003

[B53] Langille J. L. D. Rodgers W. M. (2010). Exploring the influence of a social ecological model on school-based physical activity. *Health Educ. Behav.* 37 879–894. 10.1177/1090198110367877 20980534

[B54] Lyu S. O. Lee H. (2013). Market segmentation of golf event spectators using leisure benefits. *J. Travel Tour. Market.* 30 186–200. 10.1080/10548408.2013.774913

[B55] Mardia K. V. (1970). Measures of multivariate skewness and kurtosis with applications. *Biometrika* 57 519–530. 10.1093/biomet/57.3.519

[B56] McLeroy K. R. Bibeau D. Steckler A. Glanz K. (1988). An ecological perspective on health promotion programs. *Health Educ. Q.* 15 351–377. 10.1177/109019818801500401 3068205

[B57] Misener L. Taks M. Chalip L. Green B. C. (2015). The elusive “trickle-down effect” of sport events: assumptions and missed opportunities. *Manag. Sport Leisure* 20 135–156.

[B58] Mossey J. M. Shapiro E. (1982). Self-rated health: a predictor of mortality among the elderly. *Am. J. Public Health* 72 800–808. 10.2105/ajph.72.8.800 7091475 PMC1650365

[B59] Mullin B. J. Hardy S. Sutton W. A. (2014). *Sport Marketing*, 4th Edn Champaign, IL: Human Kinetics.

[B60] Murray A. D. Archibald D. Murray I. R. Hawkes R. A. Foster C. Barker K. (2018). 2018 International consensus statement on golf and health to guide action by people, policymakers and the golf industry. *Br. J. Sports Med.* 52 1426–1436. 30245478 10.1136/bjsports-2018-099509 PMC6241627

[B61] Murray A. D. Daines L. Archibald D. Hawkes R. Grant L. Mutrie N. (2017a). The relationship and effects of golf on physical and mental health: a scoping review protocol. *Br. J. Sports Med.* 50 647–650. 10.1136/bjsports-2015-095914 27130924

[B62] Murray A. D. Turner K. Archibald D. Schiphorst C. Griffin S. A. Scott H. (2017b). An observational study of spectators’ step counts and reasons for attending a professional golf tournament in Scotland. *BMJ Open Sport Exerc. Med.* 3 1–7. 10.1136/bmjsem-2017-000244 PMC5530109 28761718

[B63] Murray A. D. Hawkes R. A. Kelly P. Grant L. Mutrie N. (2019). Do golf fans walk the talk? Follow-up of spectators’ beliefs and self-reported physical activity 3 months after they attended a professional golf tournament in the UK. *BMJ Open Sport Exerc. Med.* 5 1–6. 10.1136/bmjsem-2018-000503 PMC6347860 30740236

[B64] Mutter F. Pawlowski T. (2014). Role models in sports–Can success in professional sports increase the demand for amateur sport participation? *Sport Manag. Rev.* 17 324–336. 10.1016/j.smr.2013.07.003

[B65] Olsson U. H. Foss T. Troye S. V. Howell R. D. (2000). The performance of ML, GLS, and WLS estimation in structural equation modeling under conditions of misspecification and nonnormality. *Struct. Equat. Model.* 7 557–595. 10.1207/s15328007sem0704_3

[B66] Parkkari J. Natri A. Kannus P. Mänttäri A. Laukkanen R. Haapasalo H. (2000). A controlled trial of the health benefits of regular walking on a golf course. *Am. J. Med.* 109 102–108. 10.1016/s0002-9343(00)00455-1 10967150

[B67] Pikora T. Giles-Corti B. Bull F. Jamrozik K. Donovan R. (2003). Developing a framework for assessment of the environmental determinants of walking and cycling. *Soc. Sci. Med.* 56 1693–1703. 10.1016/s0277-9536(02)00163-6 12639586

[B68] Potwarka L. R. Leatherdale S. T. (2016). The Vancouver 2010 Olympics and leisure-time physical activity rates among youth in Canada: any evidence of a trickle-down effect? *Leisure Stud.* 35 241–257. 10.1080/02614367.2015.1040826

[B69] Qian T. Y. Wang J. J. Zhang J. J. (in press). Push and pull factors in esports live-streaming: a partial least square structural equation modeling (PLS-SEM) approach. *Int. J. Sport Commun.*

[B70] Qian T. Y. Zhang J. J. Wang J. J. Hulland J. (2019). Beyond the game: dimensions of esports online spectator demand. *Commun. Sport* 1:2019.

[B71] Rantakokko M. Iwarsson S. Portegijs E. Viljanen A. Rantanen T. (2015). Associations between environmental characteristics and life-space mobility in community-dwelling older people. *J. Aging Health* 27 606–621. 10.1177/0898264314555328 25326130

[B72] Rice R. W. (1984). Organizational work and the overall quality of life. *Appl. Soc. Psychol. Ann.* 5 155–178.

[B73] Robinson M. J. Carpenter J. R. (2002). The day of the week’s impact on selected socio-demographic characteristics and consumption patterns of spectators at a LPGA event. *Sport Market. Q.* 11 242–247.

[B74] Robinson M. J. Trail G. T. Kwon H. (2004). Motives and points of attachment of professional golf spectators. *Sport Manag. Rev.* 7 167–192. 10.1016/s1441-3523(04)70049-2

[B75] Ryan R. M. Deci E. L. (2001). On happiness and human potentials: a review of research on hedonic and eudemonic well-being. *Ann. Rev. Psychol.* 52 141–166. 11148302 10.1146/annurev.psych.52.1.141

[B76] Saelens B. E. Sallis J. F. Black J. B. Chen D. (2003a). Neighborhood-based differences in physical activity: an environment scale evaluation. *Am. J. Pub. Health* 93 1552–1558. 12948979 10.2105/ajph.93.9.1552 PMC1448009

[B77] Saelens B. E. Sallis J. F. Frank L. D. (2003b). Environmental correlates of walking and cycling: findings from the transportation, urban design, and planning literatures. *Ann. Behav. Med.* 25 80–91. 12704009 10.1207/S15324796ABM2502_03

[B78] Sallis J. Bauman A. Pratt M. (1998). Environmental and policy interventions to promote physical activity. *Am. J. Prevent. Med.* 15 379–397. 10.1016/s0749-3797(98)00076-2 9838979

[B79] Sallis J. F. Cervero R. B. Ascher W. Henderson K. A. Kraft M. K. Kerr J. (2006). An ecological approach to creating active living communities. *Annu. Rev. Public Health* 27 297–322. 16533119 10.1146/annurev.publhealth.27.021405.102100

[B80] Sallis J. F. Owen N. Fisher E. (2015). Ecological models of health behavior. *Health Behav.* 5 43–64.

[B81] Sato M. Du J. Inoue Y. (2016a). Rate of physical activity and community health: evidence from U.S. counties. *J. Phys. Activ. Health* 13 640–648. 10.1123/jpah.2015-0399 26595939

[B82] Sato M. Jordan J. S. Funk D. C. (2016b). A distance-running event and life satisfaction: the mediating roles of involvement. *Sport Manag. Rev.* 19 536–549.

[B83] Sato M. Inoue Y. Du J. Funk D. C. (2019). Access to parks and recreational facilities, physical activity, and health care costs for older adults: evidence from US counties. *J. Leisure Res.* 50 220–238.

[B84] Sato M. Jordan J. S. Funk D. C. (2014). The role of physically active leisure for enhancing quality of life. *Leisure Sci.* 36 293–313.

[B85] Sato M. Jordan J. S. Funk D. C. (2015). Distance running events and life satisfaction: a longitudinal study. *J. Sport Manag.* 29 347–361.

[B86] Sato M. Yoshida M. Wakayoshi K. Shonk D. J. (2017). Event satisfaction, leisure involvement and life satisfaction at a walking event: the mediating role of life domain satisfaction. *Leisure Stud.* 36 605–617.

[B87] Siu N. Y. M. Wan P. Y. K. Dong P. (2012). The impact of the servicescape on the desire to stay in convention and exhibition centers: the case of Macao. *Int. J. Hosp. Manag.* 31 236–246.

[B88] Stokols D. (1992). Establishing and maintaining healthy environments: toward a social ecology of health promotion. *Am. Psychol.* 47 6–22. 1539925 10.1037//0003-066x.47.1.6

[B89] Stokols D. (1996). Translating social ecological theory into guidelines for community health promotion. *Am. J. Health Promot.* 10 282–298. 10159709 10.4278/0890-1171-10.4.282

[B90] Szymanski D. M. Henard D. H. (2001). Customer satisfaction: a meta-analysis of the empirical evidence. *J. Acad. Market. Sci.* 29 16–35.

[B91] The Golf Tournament Promotion Association of Japan (2018). *Tour Yearbook 2018.* Tokyo: GTPA.

[B92] Tsuji Y. Bennett G. Zhang J. J. (2007). Consumer satisfaction with an action sports event. *Sport Market. Q.* 16 199–208.

[B93] Tudor-Locke C. Bassett D. R. (2004). How many steps/day are enough? *Sports Med.* 34 1–8. 14715035 10.2165/00007256-200434010-00001

[B94] Wakefield K. L. Blodgett J. G. (1996). The effect of servicescape on customers’ behavioral intentions in leisure service settings. *J. Serv. Market.* 10 45–61.

[B95] Wakefield K. L. Blodgett J. G. Sloan H. (1996). Measurement and management of the sportscape. *J. Sport Manag.* 10 15–31.

[B96] Wakefield K. L. Sloan H. (1995). The effects of team loyalty and selected stadium factors on spectator attendance. *J. Sport Manag.* 9 153–172.

[B97] Wang T. R. Min S. D. Kim S. K. (2013). Fulfillment of sport spectator motives: the mediation effect of well-being. *Soc. Behav. Pers.* 41 1421–1433.

[B98] Wann D. L. Waddill P. J. Polk J. Weaver S. (2011). The team identification–social psychological health model: Sport fans gaining connections to others via sport team identification. *Group Dynam.* 15 75.

[B99] Warner S. (2019). Sport as medicine: how F3 is building healthier men and communities. *Sport Manag. Rev.* 22 38–52.

[B100] Watanabe Y. Gilbert C. Aman M. S. Zhang J. J. (2018). Attracting international spectators to a sport event held in Asia: the case of formula one petronas malaysia grand prix. *Int. J. Sports Market. Sponsor.* 19 194–216.

[B101] Watanabe Y. Matsumoto K. Nogawa H. (2013). Variables influencing spectators’ desire to stay at a professional golf tournament In Japan. *Contemp. Manag. Res.* 9 283–298.

[B102] Watanabe Y. Zhang J. J. (2019). To stay or not to stay? Japanese spectators’ event-related perspectives on a professional golf tournament. *Manag. Sport Leisure* 24 244–261.

[B103] Weed M. Coren E. Fiore J. Wellard I. Chatziefstathiou D. Mansfield L. (2015). The Olympic Games and raising sport participation: a systematic review of evidence and an interrogation of policy for a demonstration effect. *Eur. Sport Manag. Q.* 15 195–226.

[B104] Zeithaml V. A. Berry L. L. Parasuraman A. (1996). The behavioral consequences of service quality. *J. Market.* 60 31–46.

[B105] Zhang J. J. Lam E. T. C. Connaughton D. P. (2003). General market demand variables associated with professional sport consumption. *Int. J. Sports Market. Spons.* 5 33–55.

[B106] Zhang J. J. Lam E. T. C. Connaughton D. P. Bennett G. Smith D. W. (2005). Development of a scale to measure spectator satisfaction toward support programs of minor league hockey games. *Int. J. Sport Manag.* 6 47–70.

[B107] Zhang J. J. Pease D. G. Hui S. C. Michaud T. J. (1995). Variables affecting the spectator decision to attend NBA games. *Sport Market. Q.* 4 29–39.

